# Challenges in Permeability Assessment for Oral Drug Product Development

**DOI:** 10.3390/pharmaceutics15102397

**Published:** 2023-09-28

**Authors:** Mirko Koziolek, Patrick Augustijns, Constantin Berger, Rodrigo Cristofoletti, David Dahlgren, Janneke Keemink, Pär Matsson, Fiona McCartney, Marco Metzger, Mario Mezler, Janis Niessen, James E. Polli, Maria Vertzoni, Werner Weitschies, Jennifer Dressman

**Affiliations:** 1 NCE Drug Product Development, Development Sciences, AbbVie Deutschland GmbH & Co. KG, 67061 Ludwigshafen, Germany; 2 Drug Delivery and Disposition, Department of Pharmaceutical and Pharmacological Sciences, KU Leuven, 3000 Leuven, Belgium; 3 Chair of Tissue Engineering and Regenerative Medicine, University Hospital Würzburg, 97070 Würzburg, Germany; constantin.berger@uni-wuerzburg.de; 4 Department of Pharmaceutics, University of Florida, 6550 Sanger Road, Orlando, FL 32827, USA; 5 Department of Pharmaceutical Biosciences, Uppsala University, 75124 Uppsala, Swedenjanis.niessen@uu.se (J.N.); 6 Roche Pharma Research and Early Development, Roche Innovation Center Basel, F. Hoffmann-La Roche AG, 4070 Basel, Switzerland; janneke.keemink@roche.com; 7 Department of Pharmacology and SciLifeLab Gothenburg, University of Gothenburg, 40530 Gothenburg, Sweden; par.matsson@gu.se; 8 School of Veterinary Medicine, University College Dublin, D04 V1W8 Dublin, Ireland; fiona.mccartney@ucd.ie; 9 Translational Center for Regenerative Therapies (TLZ-RT) Würzburg, Branch of the Fraunhofer Institute for Silicate Research (ISC), 97082 Würzburg, Germany; 10 Quantitative, Translational & ADME Sciences, AbbVie Deutschland GmbH & Co. KG, 67061 Ludwigshafen, Germany; mario.mezler@abbvie.com; 11 Department of Pharmaceutical Sciences, University of Maryland, Baltimore, MD 21021, USA; jpolli@rx.umaryland.edu; 12 Department of Pharmacy, National and Kapodistrian University of Athens, 157 84 Zografou, Greece; vertzoni@pharm.uoa.gr; 13 Institute of Pharmacy, University of Greifswald, 17489 Greifswald, Germany; 14 Fraunhofer Institute of Translational Medicine and Pharmacology, 60596 Frankfurt, Germany

**Keywords:** permeability, drug absorption, oral drug delivery, in vitro, in silico, in vivo

## Abstract

Drug permeation across the intestinal epithelium is a prerequisite for successful oral drug delivery. The increased interest in oral administration of peptides, as well as poorly soluble and poorly permeable compounds such as drugs for targeted protein degradation, have made permeability a key parameter in oral drug product development. This review describes the various in vitro, in silico and in vivo methodologies that are applied to determine drug permeability in the human gastrointestinal tract and identifies how they are applied in the different stages of drug development. The various methods used to predict, estimate or measure permeability values, ranging from in silico and in vitro methods all the way to studies in animals and humans, are discussed with regard to their advantages, limitations and applications. A special focus is put on novel techniques such as computational approaches, gut-on-chip models and human tissue-based models, where significant progress has been made in the last few years. In addition, the impact of permeability estimations on PK predictions in PBPK modeling, the degree to which excipients can affect drug permeability in clinical studies and the requirements for colonic drug absorption are addressed.

## 1. Introduction

The aim of drug development is to balance activity at the target with the absorption, distribution, metabolism and excretion (ADME) properties of a drug, ensuring that the ultimate goal is achieved—the successful treatment of the disease [1,2]. For this purpose, the drug needs to be appropriately released, absorbed at the site of delivery, distributed to the site of action and throughout the body and metabolized and eventually excreted from the body, terminating its availability to mediate a function within the body.

In the past, drugs have traditionally been relatively small organic chemical entities, but recently, a broader spectrum of drug modalities, which move beyond the classical “rules of 5” (bRo5)-type chemical structure, have been introduced into the pipeline [3]. Larger chemical structures, including bifunctional degradomer molecules, like PROTACs (proteolysis targeting chimera), and smaller molecular glues [4], as well as a whole plethora of diverse biologics, ranging from monoclonal antibodies, antibody–drug conjugates (ADCs) [5], nucleotide pharmaceuticals in the form of siRNA, microRNA and aptamers to gene therapy and cell therapy approaches [6,7], require the adaptation of the methods used to determine the absorption and disposition of a drug substance in the body [8,9]. The modification of in vitro and preclinical in vivo test systems to predict whether and how a drug molecule is absorbed and distributed in the human body, as well as the formulation of the drug substance to enable it to be applied via the most appropriate route of administration, are crucial steps to the successful development of these new drug classes. For all ADME processes, particularly those including drug absorption and distribution, passive, as well as active, permeability can play an important role. In particular, the absorption of new chemical entities after oral administration is critical to bringing the drug to market, and a range of models need to be set up to predict the absorption from preclinical to clinical stages [10,11].

The ultimate goal of drug development is to achieve high drug exposures with minimal side effects at the lowest possible dose. In vitro, ex vivo and preclinical, as well as clinical in vivo, approaches can assist in the identification of the best compound for development. As active and passive transport mechanisms can be relevant to drug permeability, methods are needed that accurately reflect both processes. Regulatory guidance also stipulates the need for permeability studies, for example in BCS-based biowaivers. Further, it is increasingly recognized that novel approaches are needed in drug discovery and pharmaceutical development to reliably predict the permeability of complex organic molecules in humans. 

In this review, we summarize the current methods used to predict and describe permeability, ranging from basic principles to in vivo and in silico models, and discuss emerging methods that may drive permeability determination from early drug discovery to the market in the future.

### 1.1. Permeability across Cellular Barriers

**Figure 1:**
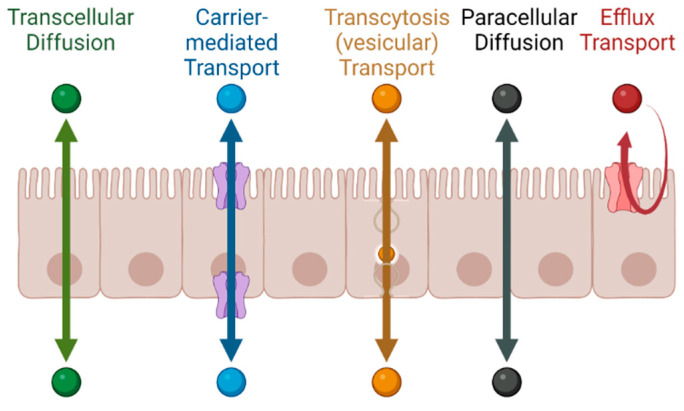
Transport processes across an epithelial cell layer.

Permeability can be defined simply as how easily a molecule crosses a biological membrane. It is expressed as the velocity—distance per unit time (e.g., in units of cm/s)—at which the molecule crosses the membrane, irrespective of whether its transport mechanism is active or passive [12]. For all molecular types described above, the relevant permeability for oral administration is across enterocyte plasma membranes or between the cells [13]. In transcellular absorption, the drug encounters the gut enterocytes and must cross into the interstitial fluid at the basolateral side of the cells to be absorbed, after which stage it eventually enters the blood stream. As described in Figure 1, permeability across a cellular barrier can occur through the cells (transcellular), during which transport can occur via transcellular diffusion; through transporter proteins (active or facilitated); either on both sides of the cell or only one side; or through cellular vesicles (transcytosis) [14]; alternatively, permeability can refer to movement between the cells (paracellular diffusion). Efflux transport, in contrast to the above-mentioned mechanisms, is a directed, protein-mediated transport out of the cell [15,16,17,18]. While transcellular diffusion, paracellular diffusion and facilitated transport are passive and follow the electrochemical gradient across the cells from the sites of higher to lower electrochemical potential, active and efflux transport can occur against the electrochemical gradient. Thus, both active uptake and efflux transport are critical components of drug ADME [19,20]. Transcytosis is mostly relevant for larger cargo, including drugs like antibodies or antibody–drug conjugates, and occurs through endosomal vesicles.

After oral dosing, the drug encounters a range of physiological conditions that influence drug absorption. For example, the pH in the gastrointestinal tract in humans ranges from pH 1 to pH 5 in the stomach, depending on prandial state, and between pH 5 and 8 in the small intestine (increasing from duodenum to ileum) and the colon [21]. As many drug substances are ionizable over at least part of this range of pH, it is important to note the interplay between solubility, which is favored by ionization, and permeability, which is favored by the non-ionized drug state. As a result of the substantial intra-individual and inter-individual variation in the physiological parameters of the GI tract [22], as well as their effects on the physicochemical properties of the drug substance, such as solid state and solubility, the pharmaceutical parameters of the formulation (type, composition, and quality of pharmaceutical excipients), as well as the manufacturing parameters, can affect drug permeability across the gut wall and, thus, absorption after oral dosing.

### 1.2. The Role of Permeability in Pharmaceutical Development

Permeability can be measured by determining the disappearance of a drug substance from the intestinal lumen (donor compartment) or measuring the appearance of a drug substance in a compartment subsequent to the semipermeable membrane in an in vitro experiment (receiver compartment). The disappearance from the intestinal lumen is quantified using the effective permeability (*P_eff_*) and the appearance of the drug substance in an in vitro receiver compartment with the apparent permeability (*P_app_*) [11,23]. 

The transport rate across the intestinal epithelium depends on the exposed surface area, the drug concentration in the gut lumen and active and passive transport parameters. *P_eff_* can, thus, be determined, for example, via perfusion in gut segments, either preclinically or clinically, according to the following equation [24]:

(1) $$P_{eff}=Q_{in}\times \frac{C_{in}-C_{out}}{C_{out}\times A}$$

where *Q_in_* is the perfusate flow rate in a system, *A* is the area available for permeation and *C_in_* and *C_out_* is the concentration entering and leaving the system, respectively. 

*P_app_* is often studied in transwell assays employing artificial semipermeable membranes or cell layers. In this setup, the movement of a drug substance from a donor compartment through the membrane into the receiver compartment is measured by sampling the receiver compartment. The permeability can be described via the following equation [11,25]:

(2) $$P_{app}=\frac{dQ}{dt}\times \frac{1}{C_{0}\times A}$$

*P_app_* depends on the appearance of the compound in the acceptor compartment over time (*dQ*/*dt*), the initial concentration of the compound in the donor compartment (*C*_0_) and the surface area of the membrane (*A*). Both permeability measures are highly relevant and used throughout the process of pharmaceutical development, as described in more detail below.

As both solubility and permeability define the penetration of a drug across a biological membrane, they need to be taken into consideration in concert. The Biopharmaceutics Classification System (BCS) has been used for many years as a guidepost for drug development [26,27]. Although the BCS can be applied to all types of compounds, further aspects will need to be considered for complex bRo5 chemical entities [8]. Structural constituents, including size, polarity, intramolecular hydrogen bonds and chameleonicity, need to be optimized and tested in relevant in vitro systems [28]. As bRo5 compounds have complex physicochemical properties, “standard” in vitro methods often do not lead to reliable results. New methods, including mucin-protected cellular models [29], or studies with co-solvents or biorelevant media may be of help [30]. 

In addition, quantitative structure–activity relationship (QSAR) methods are being developed and compared to in vitro and in vivo results [9]. In the past, in vitro Caco-2 cell assays were used to investigate the cellular permeability of compounds by predissolving them in DMSO. Nowadays, permeability assessment includes simple in vitro systems, like parallel artificial membrane permeability assays (PAMPA), and different cell types, including efflux (P-gp, BCRP fully and MRP2 and BSEP with vesicles) and uptake (OATP1B1, OATP1B3, OAT1, OAT3, OCT1, OCT2, OATP2B1, MATE1 and MATE2K) transporter studies, employing recombinant cell lines with different cellular backgrounds. Some recombinant models include cell backgrounds with reduced intrinsic efflux transporter expression to enhance the assay window and data quality [31,32,33]. To better characterize the transporters of which a drug might be a substrate, inhibitor or induction studies may also be required. 

In vivo studies can be run in wild-type rodents, but humanized transporter mouse models or transporter knockout mice can be used to supplement the experimental toolbox, usually for specific questions, such as whether a drug is able to cross the blood–brain barrier. A simplified table summarizing permeability studies at the various stages of development is shown below (Table 1). 

These studies are successively implemented and executed within a project. In the early stages of drug discovery and development, before a candidate is nominated for further development (pre-candidate nomination; pre-CN), the drug candidate is studied in high-throughput simplified methods, while at a later stage in the drug development process (post-CN), permeability is evaluated via more complex methods, including complex rodent models, as well as dynamic in silico modeling approaches, culminating in permeability studies of volunteers in clinical studies. In addition to the drug substance, the final drug product is studied at these stages. An excellent overview of the current methods is provided by O’Shea et al. [11] and described in more detail below. 

Based on the BCS system, regulatory bodies have issued harmonized system-based biowaiver guidance (ICH M9) with the intent to reduce the need for in vivo bioequivalence studies for drug products used in early clinical development through to commercialization, for line extensions of the same pharmaceutical form of innovator products, in applications for the approval of generic drug products, and for post-approval changes [26,35,36]. In this regulatory document, a scientific approach based on the aqueous solubility and intestinal permeability characteristics of drugs is used. Clear eligibility requirements for permeability according to ICH M9 include in vivo human PK studies demonstrating F_abs_ > 85 %, mass balance studies showing >85% of the drug recovered in urine (a) as the parent or (b) the sum of parent and phase 1 and/or 2 (oxidative and conjugative) metabolites. This sum may also include phase 1 and/or 2 metabolites in feces, but only if it can be shown that they had previously been absorbed. In vitro assays that must be used are clearly defined. Alternatively, data from validated, standardized Caco-2 experiments may be accepted if the validation includes a minimum of five each of high-, mid- and low-permeability reference drugs at *n* ≥ 3. The verification of cell layer integrity, absence of any efflux, justified drug concentrations, and recovery of at least 80% or a mass balance evaluation are additional requirements. High permeability is demonstrated if the apparent permeability (*P_app_*) of the test compound is ≥*P_app_* of the selected high-permeability references. Under these conditions, a drug product is eligible for a BCS-based biowaiver. 

The M9 guidance further states that for BCS Class I drugs, qualitative and quantitative differences in excipients are permitted, except for excipients that are expected to affect absorption, which should be qualitatively the same and quantitatively similar i.e., within 10.0% of the amount of excipient in the reference. For BCS Class III drugs, all of the excipients should be qualitatively the same and quantitatively similar (exceptions are described in the guidance) because low-permeability compounds are considered to be more susceptible to excipient effects on absorption. In addition, the Scale-up and Post Approval Changes (SUPAC) guidance provides recommendations for the post-approval period, when changes are made to the following aspects: (1) the components or composition; (2) the site of manufacture; (3) the scale-up/scale-down of manufacture; (4) the manufacturing (process and equipment) of an immediate-release oral formulation [37]. The guidance describes different levels of changes and parameters considered for different cases, which can be applied by the sponsor, including different dissolution protocols for documentation according to solubility and permeability.

This review provides an overview of the different methods used to predict or determine permeability at the preclinical and clinical stages of development of oral drug products. We highlight the challenges and limitations of the current standard approaches (e.g., cell-based assays, animal models, etc.) and provide an outlook on emerging methods (in silico methods, gut-on-chip, human tissue based models and novel in vivo techniques). Moreover, we demonstrate how these techniques are used to address product-related questions, such as colonic absorption, the effects of certain excipients and permeability enhancers.

## 2. Permeability in Drug Discovery and Preclinical Development

In this section, different in vitro, in vivo and in silico methods used to predict or determine permeability across the intestinal epithelium are discussed. Here, the challenges of working with cell monolayer systems and animal models, which are considered standard tools in pharmaceutical development, are highlighted. Moreover, special emphasis is put on emerging methods, such as computational approaches, gut-on-chip models and human tissue-based approaches.

### 2.1. In Vitro Models

In most cases, Caco-2 or MDCK cell monolayers are applied to obtain apparent permeability (*P_app_*) values. Caco-2 cells develop the morphologic characteristics of normal enterocytes when grown on plastic dishes or nitrocellulose filters. They form polarized monolayers, and confluence is achieved after 21 days in culture [38]. By coating the transwell inserts with extracellular matrix gels, it is possible to obtain a confluent monolayer in 3–4 days [39].

Recently, gut-on-chip and human tissue-based approaches have been developed that are expected to enable a more realistic assessment of the permeation across the gut wall.

#### 2.1.1. Experimental Challenges in Cell-Based Permeability Assays

**Figure 2:**
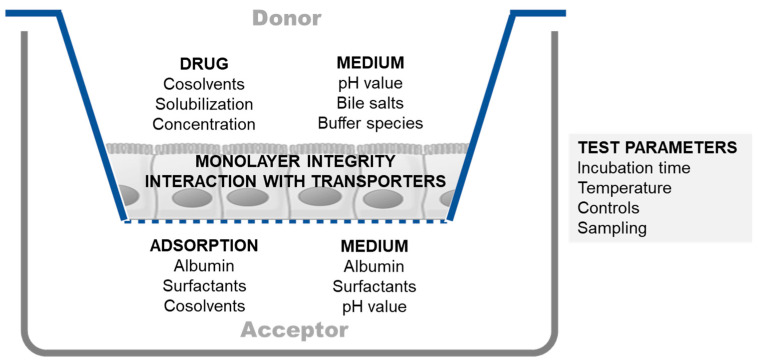
Schematic overview of frequently encountered issues and proposed solutions in cell-based permeability assays. Adapted from O’Shea et al. and Ingels et al. [11,30].

When evaluating permeability via in vitro setups, the experimental conditions selected can have a substantial impact on the final outcomes (Figure 2). Therefore, it is critical to define and control these conditions as much as possible to ensure accurate and reliable results. An excellent overview of this topic has recently been published by O’Shea et al. [11].

One particularly critical factor to consider is the selection of the solvent system used in the permeability assay, as it can affect various aspects of the experiment [30,40]. When working with contemporary drug candidates that exhibit low solubility, it is important to carefully select a solvent system that will maintain the compound of interest in solution. This may involve incorporating co-solvents or solubilizing agents to enhance solubility and prevent precipitation. Failure to adequately address solubility issues can result in the underestimation of permeability, leading to inaccurate conclusions regarding the compound’s absorption potential. 

Maintaining the integrity of the cell monolayer under the conditions used in the permeability assessment is another crucial element of cell monolayer-based studies. Using harsh solvents can easily destroy monolayer integrity and should be minimized/avoided. Nevertheless, incorporating co-solvents or solubilizing agents can reduce the adsorption of the desired compound by the materials used in the in vitro setup. Typically, adsorption is more of an issue in the receiver compartment than in the donor compartment because the concentrations in the receiver compartment are generally lower than those in the donor compartment. At this point, it is worth noting that the well plates used for growing cells are typically precoated with substances present in the cell culture medium. Precoating can help to reduce the adsorption of compounds by the well plate surface during transport experiments. Therefore, it may sometimes be advantageous not to replace the well plates with new ones when performing transport experiments.

When considering the use of solubilizing agents to increase solubility or decrease adsorption, it is important to be aware that they may reduce the free fraction and, subsequently, the permeability. This is referred to as solubility–permeability interplay [41] and should be taken into account when interpreting permeability values.

In addition to co-solvents or solubilizing agents, incorporating proteins such as albumin can decrease the adsorption of hydrophobic compounds [40]. However, the presence of albumin can lead to analytical challenges, such as interference with downstream assays. Therefore, it is important to carefully consider the trade-offs and optimize the experimental conditions when using proteins to reduce adsorption. 

When adsorption in the receiver compartment poses a challenge, an alternative solution is to focus on desorbing the compound of interest [42]. For example, after conducting a transport experiment in a Caco-2 setup, it may be possible to enhance compound recovery by removing the inserts and introducing a solvent with a stronger solubilizing capacity into the receiver compartment. Adding DMSO into the receiver compartment is one such option. 

Maintaining a concentration gradient across the cell monolayer and preventing the back flux of the tested compound are critical factors during permeability experiments. To achieve this outcome, it is necessary to limit the amount of transported compound that reaches the acceptor compartment. Typically, it is recommended to determine the permeability coefficient based on the linear part of the transport curve. If the permeation rate is too high, it can lead to significant changes in the donor concentration, which, in turn, can affect the accuracy of the results. When permeability is high, the duration of the transport experiment can be shortened to reduce the amount of substance transported and maintain sink conditions.

Up to this point, the focus has been on performing permeability assays under standard conditions. However, it is also possible to modify experimental conditions to conduct transport experiments under more biorelevant conditions. For instance, to mimic the intestinal environment, the donor compartment could be adjusted to pH 6.5, and a small amount of bile salts and lecithin could be added; in view of compatibility with a Caco-2 monolayer, the use of Fasted State Simulated Intestinal Fluid (FaSSIF) is commonly accepted as an apical solvent system [43]. Meanwhile, the receptor compartment’s pH can be adjusted to 7.4, reflecting the blood compartment.

When incorporating biorelevant conditions, it is crucial to exercise caution when interpreting results for ionizable compounds. For instance, with a basic compound, the partitioning behavior depends on the ionization status (higher ionization leads to greater solubility in the apical compartment but reduced permeation). While this approach enhances biorelevance, the pH gradient may create a situation that resembles the involvement of an efflux mechanism. When investigating the role of transporters, it is, therefore, essential to ensure an equal pH in both compartments to avoid generating “false efflux” outcomes. For example, in a study of the bidirectional transport of atenolol and metoprolol using the Caco-2 system, apical-to-basolateral and basolateral-to-apical transport were comparable for both compounds under the no pH gradient condition [44]. However, when a pH gradient was introduced (lower apical pH of 6.0 versus basolateral pH of 7.4), a shift in passive transport due to the uneven distribution of the uncharged drug species led to a “false” efflux ratio (asymmetry in transport was observed).

The outcome of an experiment can be significantly influenced by specific interactions and mechanisms that are present under different experimental conditions. Factors such as the choice of buffer species, modulation of the activity of transporters, and temperature at which the experiment is conducted can all affect the results. The following two case studies illustrate this point.

The selection of the appropriate buffer species is crucial to the permeability measurement of fosamprenavir. Fosamprenavir, a phosphate ester prodrug, was developed to address the solubility issues of its parent compound amprenavir. Due to its high solubility in the intestinal environment, it is rapidly absorbed following conversion into amprenavir by intestinal alkaline phosphatase. However, when investigating the permeation of fosamprenavir in the Caco-2 system using FaSSIF as the apical solvent system, no transport was observed. It appeared that the inorganic phosphate used as a buffer species in FaSSIF inhibited alkaline phosphatase activity [45]. Thus, to obtain reliable permeation results for amprenavir using fosamprenavir as a prodrug, an alternative buffer species must be used to replace phosphate.

A second case that emphasizes the importance of experimental conditions is related to the influence of solvent system components on the transport characteristics of the substrates of the efflux transporter P-gp [43]. The activity of these transporters can be modulated via the addition of endogenous bile salts and/or excipients that are present in drug products. The inclusion of these compounds in the solvent system may result in a reduction in the asymmetry of apical-to-basolateral versus basolateral-to-apical transport. This issue can mask the effect of P-gp. However, since these compounds are also present in the intestinal environment, they may actually better reflect the real transport characteristics and enhance the biorelevance of the permeability assay.

The reasoning presented highlights the significance of considering the experimental conditions involved in enabling a meaningful comparison of results when conducting permeability experiments using an in vitro cell culture setup. It is also important to develop a protocol, taking into account the goal of this study (initial permeability ranking versus biorelevant permeability assessment versus mechanistic studies). Various factors, such as transport buffer composition, pH, transport temperature and time, the inclusion of co-solvent or solubilizing agents, the creation of sink conditions, and the sampling method used, need to be taken into account to ensure the reliability of the transport results [30]. The influence of the medium on monolayer integrity should not be significant. Biorelevant conditions (e.g., pH, bile salts, and food components) may affect permeability directly (for example, bile salts may be influencing P-gp functionality) [46] or indirectly through the alteration of the free fraction (the so called solubility–permeability interplay) [47]. Only when carefully considering all experimental conditions can precise and reliable permeability data be obtained.

#### 2.1.2. Gut-on-Chip Models

Microfluidic gut-on-chip models (GoC) are an emerging tool used for modeling in vitro physiological and pathophysiological processes in the intestine. These models provide a constant flow of oxygen and nutrient supply while removing waste residues, resulting in a physiologically relevant environment. Under these conditions, intestinal cells spontaneously differentiate and form 3D villi-like structures with brush borders, tight junctions and mucus layers closely mimicking the human intestine [48,49]. GoC have generally been designed based on (i) the traditional transwell system [50,51,52], using a circular porous membrane, or (ii) a semi-permeable porous membrane separating two sides of a linear channel or tube [53,54]. Membranes are typically seeded with gut epithelial cells, often Caco-2 cells potentially co-cultured with HT29 cells. Alternatively, primary cells derived from the human or animal gut can be incorporated, especially for modeling specific disease phenotypes or showing improved physiological relevance [55]. The cells are allowed to adhere for some time, after which period media are pumped alongside the cell layer using a syringe or a peristaltic pump [48,56]. In some cases, the microfluidics are gravity driven, e.g., in the OrganoPlate^®^ from Mimetas (Oegstgeest, The Netherlands), which is placed on a rocker [53]. Depending on the application, endothelial or immune cells can be grown on the basolateral side of the membrane, and/or elements of the human microbiota can be added to further recapitulate the intestinal environment [57,58]. Furthermore, peristaltic motions have been created by adding pressure pulses [56]. Generally, GoC can be accessed from both the apical and basolateral side to measure barrier function. Membrane integrity is often evaluated using transepithelial electrical resistance (TEER) [56,59] or paracellular permeability measurements with fluorescently labeled dextran [53,56,60]. 

Although many GoC systems are available, few have been used to study the permeability of (small molecule) drugs. One of the transwell-based microphysiological systems (MPS) proposed for drug permeation studies is the CN Bio system (Cambridge, UK) using Caco-2 and HT29 cells. In this system, the GoC can be coupled to a chip mimicking other organs (e.g., the liver) to capture the interplay between multiple organs. In addition, it has been shown that this GoC model allows the evaluation of carboxylesterase- and CYP450-mediated gut metabolism [52,61]. Santbergen et al. also used a Caco-2/HT29 co-culture in a transwell-based system and coupled it to a chip-based LC-MS setup to facilitate the bioanalysis of small molecules and improve throughput. For verapamil, their system showed similar results to those of a static transwell system, whereas for ergotaminine, a difference was observed. The authors suggested that shear stress affects the permeability of this compound, indicating the importance of dynamic systems [50]. However, no correlation was made with the in vivo situation. Amirabadi et al. established a MPS system using porcine or human colon tissue instead of cells and were able to distinguish between paracellular and transcellular transport. The setup allowed the rank ordering of compounds according to permeability but, unfortunately, did not directly correlate with the fraction absorbed in humans; a potential reason is that the MPS did not capture permeation in the entire gastrointestinal tract but only did so in the colon [51]. Sasaki et al. used a channel-based MPS to evaluate intestinal permeability, as well as carboxylesterase-mediated metabolism. They observed that the permeability depended on the presence of albumin, which was added to avoid non-specific binding, improve sink conditions and aid the solubilization of lipophilic drugs [54]. As pointed out in Section 2.1.1., non-specific binding is an important factor to consider when using gut/organ-on-chip devices in pharmaceutical applications, as it reduces free drug concentrations in assays where small amounts of drugs need to be detected [62]. ABCB1 (P-gp) and ABCG2 (BCRP) mRNA expression was found to be significantly lower in the microfluidics devices than in the transwells. However, there was similar P-gp functionality in both. Yeon and Park determined the permeability of 10 drugs in a microhole-trapped Caco-2 based MPS. To the best of our knowledge, this study is the only paper that showed a good correlation between permeability in a GoC model and in vivo permeability in human (R^2^ = 0.90) and rat (R^2^ = 0.88) models, as well as the fraction absorbed in humans (R^2^ = 0.96). Although the correlations found were good, similar or better correlations were found using (modified) static Caco-2 cultures [60,63,64]. 

The reported studies have suggested the potential for using GoC devices in drug permeability studies; however, only a limited number of compounds have been investigated to date, and feasibility has yet to be systematically evaluated using a large set of compounds with diverse physicochemical properties representing a wide range of fractions absorbed in vivo. Currently, the relatively high costs of these complex and labor-intensive systems with low-to-medium throughput limits their application in permeability screening in an industrial environment [49]. However, GoC devices allow a faster differentiation of cells and more physiologically relevant conditions in terms of morphology and gene expression compared to static cultures [49]. Moreover, the potential to co-culture with multiple cell types and/or microbes provides physiologically relevant conditions. These systems can, therefore, potentially be used for mechanistic studies answering project specific questions, e.g., (i) the disposition of prodrugs, (ii) region or disease-specific permeability, (iii) permeability of new modalities that require the complex physiology of differentiated cell types or (iv) prediction of oral absorption of drugs using advanced drug delivery systems [65].

#### 2.1.3. Human Tissue-Based Models

**Figure 3:**
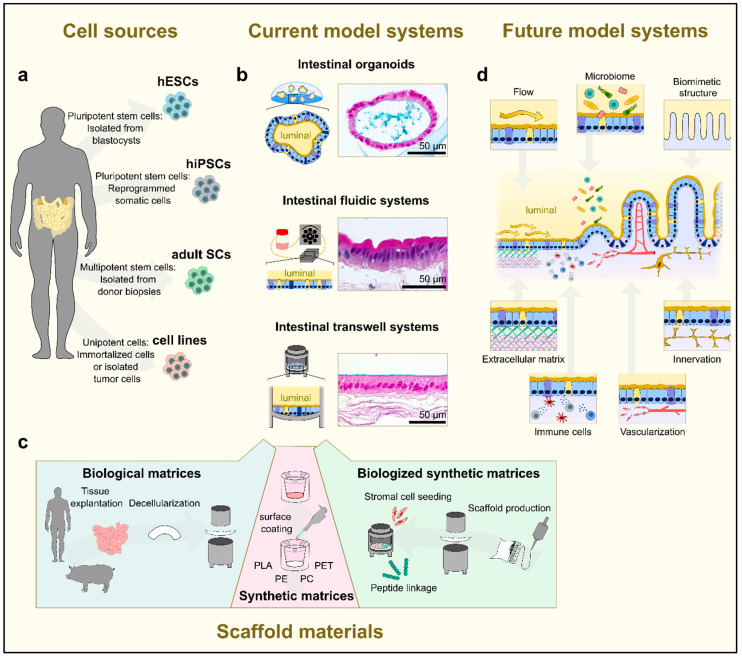
Designing human tissue-based in vitro models of the gut. (**a**) Potential cell sources used for the generation of tissue-based in vitro models. Prior to use as cells for intestinal modeling, hESCs and hiPSCs require differentiation towards the intestinal lineage, while SCs require a final differentiation to produce the individual intestinal cell types. (**b**) Schematics of currently applied culture systems i.e., intestinal organoids, fluidic and transwell-like systems; corresponding histological cross-sections of intestinal cell layer (Alzian blue [upper and lower picture] and H&E [center picture] stainings) are shown. (**c**) Summary of most commonly used scaffold materials in tissue engineering, including decellularized gut segments and synthetic matrices either coated with biomolecules, e.g., laminins or biofunctionalized with peptides or relevant niche cells. (**d**) Overview of important factors that need to be considered in future for designing even more physiologically relevant small intestinal in vitro models. Relevant components are (1) the extracellular matrix scaffolds used to stimulate ECM-regulated cell functions; (2) flow used to apply mechanical stimuli and induce mechanoresponsive signals; (3) microbiome used to consider the diverse microbial effects, such as bacterial metabolites; (4) immune cells used to enable epithelial–immune cell crosstalk, especially in terms of pathogen invasion; (5) perfusable vascular structures used to allow the efficient transport of gases, nutrients and metabolic products; (6) a biomimetic structure used to establish local tissue niches, such as the stem cell crypt; and (7) innervating structures used to reproduce neural signals regulating intestinal functions.

Human intestinal in vitro models (Figure 3) play an increasing role in predicting and evaluating pharmacokinetic properties (in particular oral bioavailability) [66]. Several endpoints have been established to validate the performance of the individual intestinal barrier function. These endpoints include the measurement of transepithelial electrical resistance (TEER), permeability of epithelial cells, changes in the gene expression of cell junction proteins and cell type-specific differentiation genes, immunotoxicology and cell proliferation. Typically, apical and basal supernatants, as well as cell lysates, are used to monitor compound concentrations, signaling molecules, activation factors and numerous other parameters. While previously used immortalized cells, which mostly originate from tumors and may, therefore, not be representative of the true physiological environment, offer many advantages in terms of handling, standardization and cost-efficient implementation in the preclinical stage, the extrapolation of data generated with these cell lines to in vivo conditions is often questionable. This is because tumor-based models do not represent all cellular subtypes of the native intestinal epithelium and exhibit artificial gene and protein expression profiles, which do not adequately reflect the in vivo situation. 

In recent years, several exciting novel in vitro culture protocols, which enable the long-term culture of primary intestinal stem and epithelial cells in vitro as intestinal organoid structures, also known as “mini-guts”, have been published [67,68,69]. To do so, intestinal crypts obtained from human donor tissue are mostly embedded in a three-dimensional laminin- and collagen-rich extracellular matrix [70,71]. The basal lamina is also simulated using Matrigel^®^ or Collagen I hydrogels covered with culture media containing relevant niche factors derived from Wnt, EGF, BMP, and Notch signaling pathways to maintain the intestinal stem cell niche [72,73]. Within the first days of culture, crypt cells form spherical structures with a crypt-like lumen, i.e., the so-called enterospheres. Simultaneously, this culture condition enables the generation of budding protrusions from the central spherical domain of the organoid containing LGR5+ stem cells and differentiated epithelial cell types [68,69,71,74,75,76]. The spheres can subsequently expand into multilobulated enteroids mimicking the multicellular structure of the intestinal epithelium, including crypts containing stem cells and Paneth cells, as well as villus structures containing differentiating cell entities, such as absorptive enterocytes, mucus-producing goblet cells and hormone-producing enteroendocrine cells. For the specific promotion of differentiation e.g., into the secretory lineages, Notch signaling can be reduced by γ-secretase inhibitors (such as DAPT) and the omission of Wnt3A [77]. It is worth noting that the success and yield of the individual human cell culture strongly depends on the donor tissue, including age, gut region and pathology [78]. 

A patient-derived cell source is beneficial for the establishment of healthy- and disease-related biobanks, but it clearly complicates standardization and reproducibility for in vitro cell culture applications [79]. In this respect, Fujii et al. published a refined culture system based on high-throughput single-cell RNA profiling data that support cellular diversity in human intestinal organoids [76]. In addition to patient-derived organoid technologies, there are an increasing number of protocols differentiating intestinal organoids from human iPSCs using stepwise differentiation protocols [80,81]. These human iPSC-derived organoids (HIOs) are multi-layered structures that also contain the major cell types of the small intestinal epithelium; however—so far—they still represent an immature, fetal-like phenotype of the generated intestinal cell types [82]. The maturation of HIOs is only achieved after implantation in vivo or via in vitro maturation based on the co-culture of HIOs with T lymphocytes [83]. The STAT3-activating interleukin-2 has been identified as major factor for in vitro maturation that leads to the development of adult-like phenotypes [83]. 

Taken together, the overall advantage of organoids compared to traditional 2D cell culture systems lies in the fact that they possess all physiologically relevant cell phenotypes and have a self-renewing capacity i.e., can be constantly maintained in culture [76] without significant changes in the phenotype and karyotype. Additionally, they can be cryopreserved for ‘off the shelf’ use if needed [78]. However, the drawback of an organoid lies in the spherical and ‘up-side-down’ tissue morphology, which does not allow classically applied endpoint measurements, which are typically determined within a 2D Transwell^®^ culture, such as intestinal permeability and transport. Nevertheless, through constant improvements of real-time imaging systems (two-photon, spinning disc microscopy, etc.) and mathematical modeling, organoid systems are increasingly being utilized as an efficient tool to evaluate drug dynamics, such as P-gp efflux transporter-mediated drug transport [84]. Moreover, two independent groups have recently developed an intestinal organoid model from patient-derived cells and pluripotent stem cells showing reversed (i.e., apical-out) polarity, where the apical side faces the surrounding culture media and the basal side faces the lumen [85,86]. This model will offer an additional research tool to study nutrient/drug uptake in organoids.

Another option for better access in preclinical testing is the tissue engineering (TE)-based culture concept using diverse biomaterials as scaffolds to culture intestinal (primary) cell types within the widely used transwell-like systems [87,88]. The biomaterial can either be synthetic [89,90,91] (e.g., polyester (PE), polycarbonate (PC) or polyethylene terephthalate (PET)) or of biological origin [92,93,94], such as spin silk or decellularized intestinal scaffolds prepared from porcine gut. An example of the latter is small intestinal submucosa (SIS) [95,96,97]. Whereas native biomaterials are often sufficient for cell attachment and growth due to a conserved ECM structure and basal lamina contents, synthetic matrices mostly need further surface modifications either via coating with ECM-proteins or the inclusion of supporter cells such as fibroblasts, which then build an in vivo-like environment. As opposed to synthetic matrices, which might influence the diffusion and distribution of biological or chemical compounds, biological matrices resemble a 3D microenvironment and are biocompatible; however, they often fail in terms of standardization and reproducibility [98]. In contrast to organoids, transwell-like culture systems offer a large advantage in terms of providing an accessible apical and basal compartment, thus mimicking the luminal and basal sides of the small intestinal epithelium in vitro. On the other hand, transwell-like models can only be kept in culture for a certain time-frame since self-renewing stem cells are continuously lost during culture due to the still-artificial culture conditions. To also meet these demands, emerging technology concepts try to design improved culture systems that mimic natural environmental cues in vitro [99]. Those approaches include biomimetic scaffolds produced via bioprinting technologies or microfluidic devices (bioreactors, chip systems, etc.) [100,101]. For instance, Wang et al. reported the development of a microengineered collagen-based scaffold that enables the formation of a human small intestinal epithelium with key structural features, like a crypt-villus architecture and associated cell type compartmentalization [102].

Taken together, organoid cultures can be highly representative of the in vivo situation but are currently limited by their inability to provide standardized endpoint measurements that are the norm in preclinical studies. In contrast, engineered TE-based systems meet the requirements of high reproducibility and accessibility but, for now, fail to completely resemble the functioning intestine. Combining the advantages of both culture systems represents one of the main challenges for routine implementation in oral drug product development. Ideally, adapting the complexity of TE-engineered models could follow a bottom-up process via the stepwise integration of additional factors, for instance the microbiome, immune cells, vascular structures, biochemical gradients, and mechanical stimuli. In this regard, the evolution of current and new techniques in both the engineering (e.g., 3D printing, novel surface materials, etc.) and biology (e.g., microbiome, stem cell research, assembloid techniques, etc.) fields is of great interest and expected to lead to intensive interdisciplinary exchange.

### 2.2. In Silico Methods

Computational modeling of cell permeability is applied in all stages of drug discovery and development, with the tools applied varying in nature and complexity depending on the needs of the project at that stage. From lead optimization all the way to clinical development, computational tools are extensively used, for example, to translate data generated in vitro and in non-clinical species to predict human exposure, using physiologically based pharmacokinetic (PBPK) modeling and similar approaches [103]. In the early stages of drug discovery, experimental permeability data may not yet have been generated, and the focus may rather be on the prediction of permeability from chemical structures and supporting the design of new lead molecules with optimal permeability properties. Here, we focus on such chemistry-oriented permeability prediction.

Principally, the applications of such methods can be divided into two main groups: prioritizing molecules in large compound libraries based on predicted permeability properties and providing a mechanistic understanding of what is driving permeability during lead optimization. Two conceptually different computational approaches have been applied to predict and rationalize permeability to drug molecules: structure–permeability relationships, derived using machine learning methods, and physics-based simulations of permeability. 

#### 2.2.1. Machine Learning-Based Permeability Modeling

**Figure 4:**
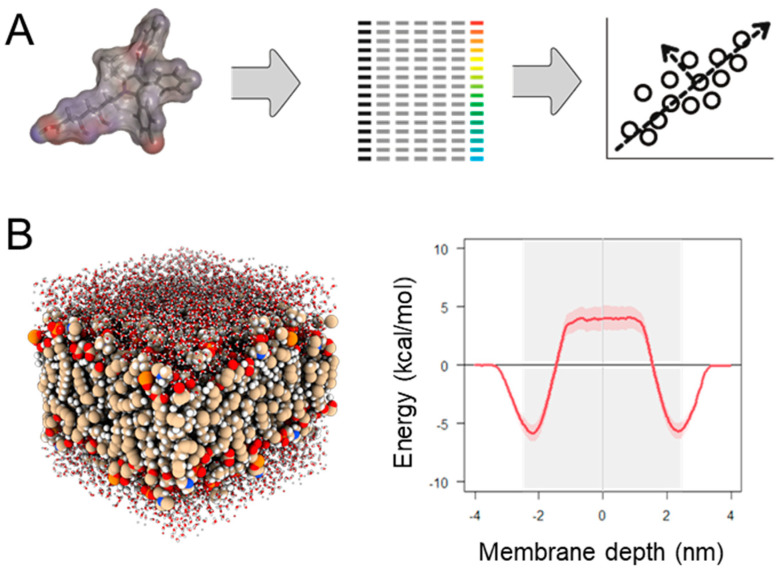
Computational methods used to estimate and rationalize membrane permeability to drug molecules. (**A**) In machine learning-based modeling of structure–permeability relationships, measured permeability values are related to a numerical description of molecular structure and properties using a machine learning algorithm. This process yields a mathematical model that can be used to predict permeability for new molecules, given that they are similar enough to molecules used in model training (‘within the applicability domain of the model’). (**B**) Molecular dynamics simulation describes movement and interactions of atoms in a system of molecules and can be used to derive energy landscapes for penetration of permeating molecules into a simulated cell membrane.

In machine learning-based structure–permeability modeling (as in any type of structure–activity relationship modeling), the measured permeabilities of a set of molecules are related to numerical descriptions of their chemical structures and/or molecular properties through a mathematical model (Figure 4A). Numerous flavors of molecular descriptors and machine learning algorithms are available and continue to be developed, and many have been explored over the years for permeability relationships. Early examples include standard linear or non-linear regression of measured permeabilities to individual descriptors, for example identifying the very often strong relationship between permeability and lipophilicity [104]. For chemically diverse, drug-like molecules, polar surface area (PSA) was shown to be an important predictor of intestinal drug permeability [105,106]. These early studies indicated that surface areas calculated via three-dimensional representations of the molecule, accounting for different molecule conformations, only marginally improved the predictions of permeability relative to using single, arbitrary conformations [107], and, hence, the more rapidly calculated 2D (or topological) TPSA was introduced and is commonly applied in permeability filters [108]. This apparent conformation-independence was likely biased by the fact that the compounds included were mostly relatively small (MW < 500 Da), aromatic and rigid, leading to relatively small variation in the exposed polar surface among conformers of the same molecule. The recent literature revisiting the role of exposed polar surface indicate that as drug molecules become larger and more flexible—entering the ‘beyond-rule-of-5′ chemical space—conformational dynamics plays an increasingly important role for membrane permeability [8,109,110].

While single-descriptor models can be informative regarding the molecular properties affecting permeation, they often do not yield useful predictions outside of series of similar molecules. Instead, combinations of large numbers of descriptors are typically used, in combination with multivariate machine learning algorithms, such as partial least squares, random forest, support vector or neural network regression [109,111,112], to better capture nuances in the driving forces of permeability in the dataset under study. Common types of descriptors are introductorily described in two excellent publications [113,114]. Importantly, the choice of descriptors and algorithms will influence both how well the model performs (i.e., how well it not only describes the molecules included in the training data, but also predicts new, unseen molecules) and how easy the results are to interpret (for example, which molecular properties or structural features are driving permeability in the dataset). While predictivity is clearly important in a structure–property relationship model, interpretability is also vital, especially if the model is used to guide the design of new molecules.

Most importantly, any machine learning model will try to describe the data with which it was provided, placing great importance on the quality of data used in training. For example, inter-laboratory variability in permeability measurements can be pronounced [115] even when (nominally) the same cell line is used. The reasons for this outcome include the clonality of the cell lines and considerable differences in assay characteristics, such as incubation times, sampling, temperature, pH, etc. Also, the fact that most experimental permeability systems—2D and 3D-cultured cells, cell-derived membrane vesicles, excised tissue and in vivo models—include multiple transport pathways that work in concert makes deriving a fully descriptive structure–permeability relationship difficult or even impossible. Despite such complexities, however, computational models may capture the dominating mechanism in a series of molecules and, thus, provide valuable mechanistic insights.

#### 2.2.2. Physics-Based Permeability Simulation

While machine learning-based structure–permeability modeling is fundamentally dependent on experimentally derived training data, physics-based techniques, such as molecular dynamics (MD) simulation (Figure 4B), can provide highly detailed information on the permeation process without the need for training data, although at a significantly greater computational cost. 

In MD, systems of molecules are simulated over time through an iterative process, where the positions of all simulated atoms are updated based on the forces imparted from the other atoms in the system. This approach yields an evolving picture of the movement of the atoms over short, typically microsecond, time scales. By placing a permeating molecule at different depths in a water/cell membrane system and calculating the free energy profile as a function of the penetration depth, the energy barriers to permeation can be calculated (Figure 4B). These energy landscapes, or ‘potentials of mean force’ (PMFs), can be integrated to yield simulated permeability coefficients. MD simulations of membrane permeability is extensively reviewed in [116]. Importantly, MD simulations also provide insights into the molecular mechanisms of membrane permeability that are difficult or impossible to experimentally study. For example, MD simulations combined with a Markov State Model defined the rate-limiting step in membrane permeation in a series of drugs, indicating that permeation was primarily limited by the transition (‘flip-flop’) between membrane leaflets for more hydrophilic molecules, whereas membrane off-rates/resolvation in water was the limiting factor for highly lipophilic molecules [117]. The transition between different conformations, which has been shown to profoundly affect the molecular properties and, hence, permeability of larger, more flexible molecules, such as macrocyclic drugs and other bRo5 molecules [8,109,110], can also be accurately simulated using MD. Recent examples are the application of MD simulation to study the permeation behavior of macrocyclic drugs [118] and cyclic peptides [119]. 

#### 2.2.3. PBPK Modeling

In physiologically based pharmacokinetic (PBPK) modeling, permeability is typically predicted via machine learning approaches or correlations between in vitro and in vivo data. Commercially available PBPK platforms, such as GastroPlus^®^ or Simcyp^®^, have built-in models to predict human jejunal effective permeability (*P_eff_*) from drug physicochemical properties and apparent permeability (*P_app_*) values obtained in in vitro Caco-2 or MDCK cell monolayers. Due to their simplicity and high-throughput compatibility, Caco-2 cells are largely used to predict human *P_eff_*. However, there are currently four challenges related to translating preclinical permeability estimates for PBPK modeling, namely (1) differences in the transcriptome, (2) the “no-micelle” assumption, (3) high lab-to-lab variability and (4) modeling segment-specific permeability. 

Even though Caco-2 cells are able to reproduce the polarized epithelium monolayer phenotype, their transcriptome significantly differs from the human duodenum transcriptome. For example, more than 1000 genes showed a more than 5-fold expression difference when comparing Caco-2 in in vitro systems and human tissue [120], so modelers should be aware that molecular mechanisms may differ between in vitro and in vivo systems. Consequently, in vivo (*P_eff_*)-in vitro (*P_app_*) drug permeability measurements correlated well for passively absorbed drugs (R^2^ = 85%). However, the permeability correlation for carrier-mediated drugs was 3- to 35-fold higher in humans above the correlation of passively absorbed drugs [120]. In fact, Caco-2 *P_app_* values led to the misclassification of four highly permeable compounds, the uptake of which is dependent on transporters [121]. The *P_app_*-*P_eff_* correlations used in the commercial PBPK models were derived using reported human jejunal effective permeability data mainly obtained from the Loc-I-Gut protocol (see Section 3.1), which only used buffer to perfuse the isolated intestinal segment [122]. Therefore, no micelles are assumed to be present in the in vivo experiment. In this context, permeability studies across Caco-2 monolayers generally use plain buffers as the medium in the donor chamber, and all resulting *P_app_*-*P_eff_* correlations are obtained under a “no micelle” assumption. However, for poorly soluble drugs, Caco-2 permeability experiments have been performed using biorelevant media in the donor chamber, which contains bile salts at concentrations above their critical micelle concentrations. Interpolating resulting *P_app_* values in *P_app_*-*P_eff_* correlation models violate their underlying assumption of “no micelle” and may lead to biased *P_eff_* values. In fact, Markopoulos et al. [123] demonstrated that *P_app_* values for hydrophobic compounds decrease upon increasing the concentration of bile salts in the donor media. For example, for danazol (logP = 4.2), *P_app_* in plain buffer is 25-fold higher than *P_app_* obtained using FeSSIF in the donor chamber [123], which significantly affects the derived *P_eff_* value (ranging from 8.2 to 0.4 × 10^−4^ cm/s when using the built-in correlation in the Simcyp Simulator v21).

Another challenge related to translating *P_app_* values is related to the very high lab-to-lab variability. This variability can be attributed to experimental aspects, as outlined above as well as to factors such as the cell passage number, cell culture conditions, number of cells or cell monolayer integrity. It can potentially mislead decisions if permeability results from different laboratories are directly compared. Lee et al. showed that Caco-2 cell permeability results for the same compounds can substantially differ between different laboratories [124]. Interpolating *P_app_* values using correlations obtained in different labs significantly increases the root mean square error associated with fraction-absorbed predictions. So, ideally, *P_eff_* or fraction absorbed should be estimated using correlation models derived in the same lab. Alternatively, *P_app_* values for high and low permeability standards can be used to calibrate external correlation models before using them to predict *P_eff_* or fraction absorbed.

When interpolating *P_app_* values in *P_app_*-*P_eff_* correlation models, we obtained estimates of the human jejunal effective permeability, since these are the in vivo data used to build such correlations [122]. In the Simcyp^®^ Simulator, users may leverage the Mech*P_eff_* model, which predicts regional differences in intestinal permeability. The Mech*P_eff_* model is based largely upon the structure described by Sugano et al. [125]. In brief, the model:

1. Handles intrinsic transcellular permeability according to the pH partition hypothesis (but allows the user to select an additional model permitting transcellular ion permeation);
2. Considers paracellular permeability separately whereby molecular size in relation to pore size is considered (via a Renkin function), in addition to pore charge–charge interactions (electrolytes can pass through the paracellular pathways);
3. Includes consideration of the luminal Unstirred Boundary Layer (UBL), which may be the rate-limiting barrier for otherwise highly permeable drugs.

The Mech*P_eff_* model can be calibrated against Jejunal-1 *P_eff_* values predicted using any of the in vitro cell permeability or in silico methods or directly determined using, for example, the Loc-I-gut method. If it is assumed that transcellular permeability dominates (rather than paracellular or unstirred boundary (mucus) layer permeability), then the on-screen P_trans,0_ (intrinsic transcellular permeability) value can be manually adjusted to reproduce the desired Jejunal-1 *P_eff_* value.

### 2.3. In Vivo Models

Animal intestinal absorption models are fundamental in many stages of the pharmaceutical development of orally administered drugs and drug products. In preclinical development, they are used to ensure adequate plasma exposure at high oral doses as part of toxicokinetic evaluation [126]. In later stages of drug development, animal intestinal absorption models are used to assess the effects of a range of biopharmaceutical properties, including the particle size and physical form of the drug, excipients, immediate- vs. modified-release formulations, enabling formulations, prandial state and gastric pH [127]. The choice of animal model (e.g., rodent, dog, pig, etc.) relies on the research question, as well as the size of the drug formulation. When evaluating intestinal absorption, drug concentrations in relevant body compartments over time are typically quantified following oral dosing. This approach means that the possibility of specifically studying intestinal drug permeability is limited by difficulties in differentiating permeability from other absorption processes. There are, however, methods that allow more detailed mechanistic studies of intestinal drug permeability in research animals. Two key animal models, namely single-pass intestinal perfusion (SPIP) and intraintestinal dosing/instillation, are presented below and in Table 2, including their advantages, disadvantages and applications.

#### 2.3.1. Single-Pass Intestinal Perfusion

**Figure 5:**
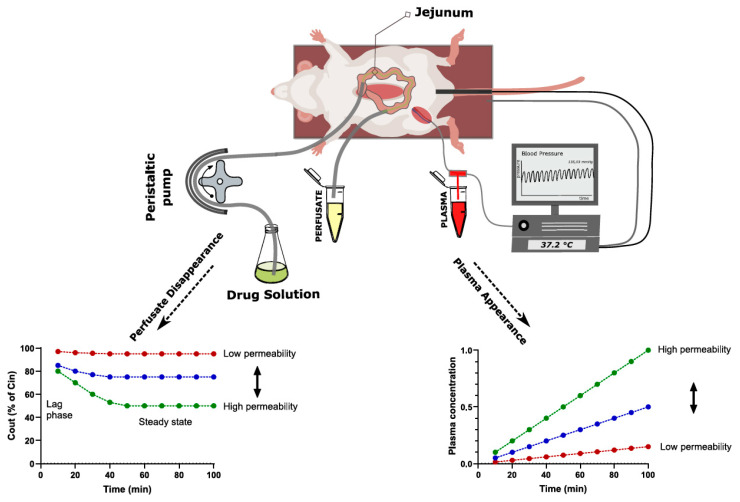
Illustration of the experimental setup of the rat single-pass intestinal perfusion model used for determining intestinal drug permeability of a test substance (blue line) and two idealized examples (green and red line) of how output data look in perfusate samples and plasma. Drug permeability can be directly determined from luminal drug disappearance (**bottom left**) or indirectly determined from plasma drug appearance (**bottom right**).

For the determination of intestinal drug permeability (and flux), a range of in situ models with small variations can be applied. They all rely on the monitoring of drug transport from, or to, an isolated intestinal segment, whereby the segment can be either a closed loop or perfused [128]. The most commonly used one is the SPIP model (Figure 5) [129]. 

In this model, effective drug permeability (*P_eff_*) is quantified by comparing the concentration of a drug solution entering (*C_in_*) and leaving (*C_out_*) a perfused intestinal segment, corrected for water flux, to the intestinal surface area of the perfused segment (*A*) and the perfusion rate (*Q*) using Equation (3).

(3) $$P_{eff}={\frac{Q}{A}}_{}\times (-\ln\left(\frac{C_{out}}{C_{in}}\right)$$

Alternatively, a deconvolution method can be applied [130], where permeability is determined by calculating an intestinal absorption rate from the perfused intestinal segment based on the drug appearance rate in plasma, the perfused luminal surface area (*A*) and the average luminal drug concentration (*C*) using Equation (4).

(4) $$P_{eff}=\frac{absorption\;rate}{A\times C}$$

This deconvolution–permeability method requires an intravenous reference of the same drug, as well as an assumption/approximation regarding the first-pass extraction in the gut wall and liver, for which we need to make corrections. 

Calculation based only on drug disappearance in the perfusate (Equation (1)) is easier from a bioanalytical and practical point of view, while the deconvolution method has a higher accuracy for drugs with low absorption [131]. The higher accuracy of the deconvolution approach is because the difference in the concentration entering and leaving (*C_in_* and *C_out_*) the perfused intestinal segment can be insignificant for low absorption drugs, while the drug concentration differences in plasma may still be substantial. However, for very low permeability drugs such as peptides, the plasma concentrations often end up being below the lower limit of quantification for the conventional LC/MS-MS methods. When plasma concentrations are too low, drug appearance in the mesenteric veins that drain the perfused segment can be sampled instead [132], thus avoiding dilution in the central blood compartment, as well as first-pass hepatic metabolism. However, this specialized method requires a blood reservoir. 

The SPIP model is very versatile, as it enables mechanistic investigations of drug permeability at defined luminal conditions. This includes the effect of, for instance, the luminal drug concentration [133], pH [134], perfusion rate [135], fasted and fed conditions [136], formulation and excipients effects [137] and regional intestinal differences [138]. It also has the advantage of maintained physiological regulation of gut functions, including intact neurohormonal and immunological feedback mechanisms, which opens up the possibility of evaluating drug permeability at different physiological and pathophysiological states [139]. Maintained physiological regulation is also a big advantage of the SPIP permeability model (and other in vivo models) compared to in vitro systems (e.g., Caco-2 cells and Ussing chamber). 

Another important advantage of in vivo permeability models over in vitro systems is the high permeability value that is generated. For instance, the permeability values of atenolol and metoprolol are 10–100 times higher in the rat intestine in vivo [129] compared to when it is mounted in the Ussing chamber [140] or compared to Caco-2 cells [141]. This result means that a higher flux (amount/time/area) is generated, which is necessary for correctly evaluating many biopharmaceutical effects, such as luminal dissolution, precipitation and supersaturation.

#### 2.3.2. Intraintestinal Dosing

**Figure 6:**
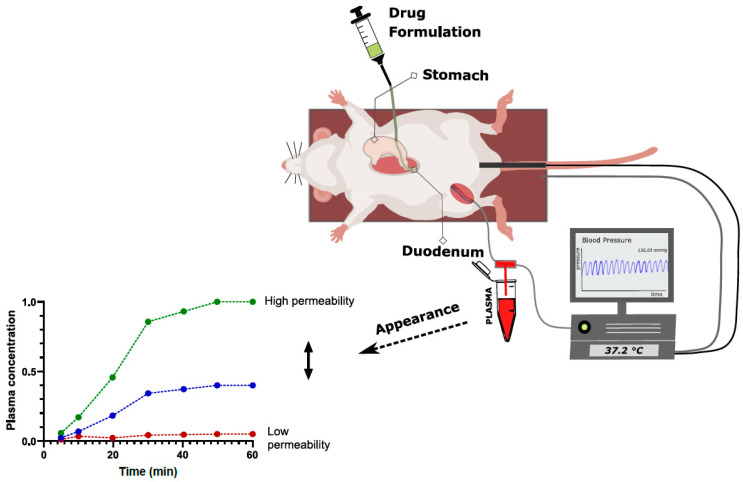
Illustration of the experimental setup of the rat intraintestinal bolus (instillation) model used for determining intestinal drug permeability and one idealized example of how output data look in plasma for a test substance (blue) and two reference substances with either high (green) or low (red) permeability. Drug permeability is determined from plasma drug appearance (bottom left).

Intraintestinal dosing of drug solutions (or formulations) directly into the intestinal lumen of suitable animal species can be used to determine permeability based on plasma drug appearance (Figure 6), using the same permeability calculation method as that described in Equation (2) [142]. 

Compared to the SPIP model, this method needs to be modified regarding intestinal surface area, as there is no defined perfused segment. The most accurate assumption is to calculate with a surface area corresponding to a water plug with an area defined by the dosed volume [143]. The methods of dose administration are plenty and include oral and anal tubing [144], stoma models [145], and luminal entry across the serosal side following laparotomy [142]. The latter method is the easiest from an experimental point of view, but the most suitable approach will rely on the species at hand and the intestinal segment to be investigated.

A major advantage of the intraintestinal bolus model for the determination of drug permeability lies in the high throughput compared to SPIP, as gastrointestinal surgery, perfusion tubing and pumps can be avoided. The method may also be used to assess the impacts of more in vivo relevant biopharmaceutical processes affecting intestinal drug absorption kinetics, such as dissolution, precipitation, gastric emptying and transit. This is also a reason why the intraintestinal bolus model is less applicable than the SPIP model for mechanistic absorption studies, as the relative impact of the different factors can be difficult to assess in detail.

## 3. Permeability in Clinical Development

### 3.1. In Vivo Methods Used to Determine Permeability in Clinical Development

**Figure 7:**
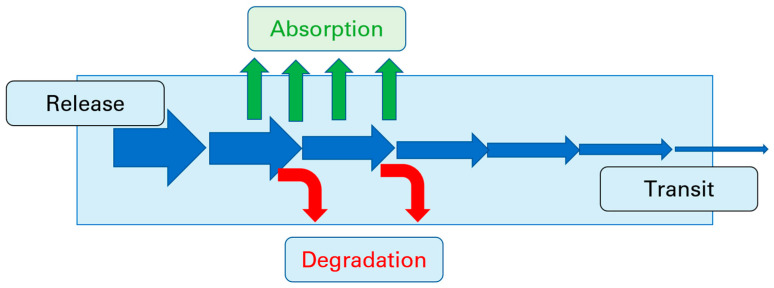
Processes that influence the fraction absorbed after oral administration.

Permeability can be defined as the ability of a substance to pass the barrier formed by the enterocytes in the GI tract, or in other words, the fraction absorbed. Due to the complexity of the processes that take place during the passage of a substance from the lumen of the gastrointestinal tract, it is, in principle, very difficult to isolate the permeability from data obtained in a clinical study. Similar to the situation in animal studies, factors such as the dissolution/release of the drug from the dosage form, drug solubility, luminal stability and intestinal transit time can all influence the fraction absorbed [10], as shown in Figure 7.

**Figure 8:**
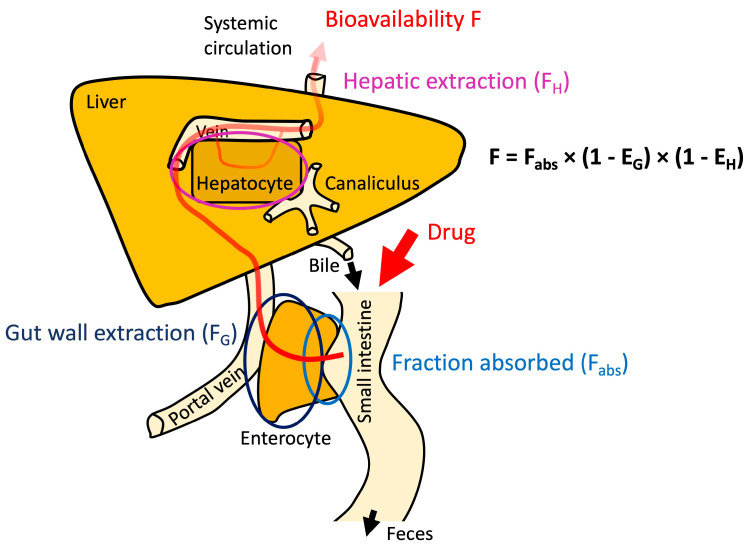
Influence of fraction absorbed, gut wall extraction and hepatic extraction on oral bioavailability.

The most obvious method of investigating permeability is to determine the absolute bioavailability after oral administration. However, the determination of the absolute bioavailability requires using intravenous administration as the reference value. This approach, in turn, requires the availability of a parenterally administrable dosage form, which for poorly water-soluble drugs may be difficult to achieve. Furthermore, absolute bioavailability is only a reliable measure of the permeability of the gastrointestinal tract if it is high. Otherwise, mechanisms such as first-pass metabolism in enterocytes or the liver, hepatic extraction or even the activity of intestinal efflux transporters may result in low bioavailability, despite a high fraction being absorbed i.e., successful negotiation of the enterocyte barrier. Most of these aspects are depicted in Figure 8. 

The determination of the absolute bioavailability can also be helpful to understanding whether the formulation influences the processes involved in oral absorption, although it may be difficult to tease out exactly which processes have been affected. 

A clinical alternative to determining absolute oral bioavailability as a measure of fraction absorbed is to conduct a mass balance study [146]. The advantage of a mass balance study is that metabolites formed after absorption can also be taken into consideration and, thus, a clearer picture of the fraction absorbed can be achieved. Another clinical alternative is microdosing [147], which can be used either in absolute bioavailability studies or mass balance studies, although intravenous dosing seems to be rather rarely performed.

The gold standard for the determination of human GI permeability according to current guidelines [26,36] is regional intestinal perfusion, which is usually performed using the Loc-I-gut method. An overview of the different methods used can be found in Sjögren et al. [127]. In principle, the methods of determining regional intestinal permeability *P_eff_* are based on determining drug disappearance from the lumen of the perfused segment at a given perfusion rate. The perfused surface area required for the calculation of *P_eff_* is determined as the surface of the perfused cylinder given by the length (l) and radius (r) of the perfused intestinal segment. For the jejunum, r = 1.75 cm is typically assumed [148]. It has to be kept in mind that *P_eff_* is a measure of the regional permeability rate, reflecting the balance between the uptake (absorption) and efflux of parent drug within the investigated segment during the experimental time frame. *P_eff_* is, therefore, not necessarily descriptive of the fraction absorbed. The advantages and limitations of intestinal perfusion techniques are discussed in a recent review [149]. To date, almost all regional perfusions in humans have been performed in the upper small intestine, as it is difficult and time-consuming to intubate subjects down to the distal small intestine, and the method is not feasible at the colonic level. 

In addition to regional perfusion techniques, it is possible to measure the regional relative bioavailability. Over the years, many swallowable devices have been used to gather information about pathophysiology in the gastrointestinal (GI) tract, and several devices have been marketed with the aim of better understanding drug absorption after oral administration.

In the pharmaceutical arena, a popular swallowable device is the Heidelberg capsule, first developed by Prof. H. G. Noller at the University of Heidelberg fifty years ago [150] which has been used to explore the pH profile in the GI tract and imbalances in the GI pH due to disorders such as gastric ulcer, hypochlorhydria and cystic fibrosis, as well as to determine the gastric emptying time of large, non-disintegrating objects [151,152,153,154].

Later, further swallowable devices were developed to monitor further GI parameters, such as temperature and pressure, in addition to the pH. One such product is the SmartPill^TM^ from Medtronic (Dublin, Ireland), which is marketed as a Motility Test System consisting of a swallowable capsule that can measure pressure, pH, transit time and temperature as it moves through the GI tract. In the clinic, the SmartPill^TM^ is used to test whether the GI motility is functioning well by measuring the gastric emptying time, transit through the colon and transit time through the intestines as a whole, as well as the pressures that are developed in the antrum and duodenum [155]. An example of its use in pharmaceutical research is the study by Koziolek et al. of intragastric pH and pressure profiles after ingestion of the high-caloric, high-fat meal used for food effect studies [156].

Concurrently, capsules were developed to investigate the ability of drugs to penetrate through the gut wall at various points within the GI tract. Although, as mentioned above, the Loc-I-Gut and related techniques have been successfully used to determine *P_eff_* in humans, as described by O’Shea et al. in their excellent summary of permeability models [11], there are many challenges associated with intubating subjects to make such measurements, and in recent years, few data have been generated via techniques relying on multi-channel intubation. Thus, analogous to the development of diagnostic capsules to replace endoscopy (preparation of the GI tract prior to intubation, the intubation procedure itself, time taken to reach more distal regions, like the ileum, etc.), devices which can be swallowed without requiring intubation became desirable for studying permeability [157]. 

One such device, the IntelliCap, was originally developed by Medimetrics (Eindhoven, The Netherlands) as an electronically controlled device for local drug delivery, but it was later primarily used as a clinical research tool to specifically study permeability. This system uses the gastrointestinal pH profile to identify the capsule’s location within the human GI tract [158,159]. In an initial study, Becker et al. explored the use of the IntelliCap to investigate diltiazem permeability in the colon. The release pattern of the diltiazem was adjusted to match that of a sustained release product containing diltiazem, and in vivo results demonstrated that the IntelliCap was able to match the pharmacokinetics of the product [160]. Söderlind et al. attempted to validate the use of the IntelliCap by studying metoprolol release in humans according to two different patterns. The first was linear release over 4 or 6 h, and here the data for the programmed and in vivo release pattern were in excellent agreement. However, when two separate pulses of metoprolol were programmed, the second pulse only released about half of the intended amount of metoprolol in the colon [158]. Another system based on a similar concept is the InteliSite system (Scintipharma, Lexington, KY, USA), which uses gamma scintigraphy to determine the position of the device in the GI tract [158,159]. Despite the mostly encouraging results generated via these devices, both the IntelliCap and InteliSite products appear to have been discontinued.

Another apparatus that can measure permeability at different locations in the GI tract is actually a hybrid of an intubation method and a device used to deliver the drug at predetermined sites [161]. Intubation is performed via a thin, nasally introduced tube to which a capsule is attached to enable the local instillation of substances in solution or solid form, e.g., as pellets at defined intestinal locations. Using this system acquired from BioPerm AB (Lund, Sweden), studies of local bioavailability, metabolism, active transport, and interactions in the small bowel, as well as in the colon, can be undertaken (www.bioperm.se, accessed on 13 April 2023). A limitation of the release device is that it is “tethered” via the intubation tube, such that the length of the tubing, together with scintigraphic data, are used to locate the position of the device within the GI tract. Two recent papers describing the pharmaceutical applications of the BioPerm method are Dahlgren et al. [162] and Hofmann et al. [163]. 

The current state of the art demonstrates a clear need for a programmable, swallowable device to measure the permeability of drugs at different locations within the GI tract. At Fraunhofer, a major project to develop a device of this type that uses sensor information rather than a tether to locate the device within the GI tract is underway. An important aspect of this development is making the accrual of data for human *P_eff_* values possible for a wider range of compounds than had been possible with the Loc-I-Gut technique. Such an expanded data bank would be extremely useful for benchmarking permeability studies based on cell lines and estimations of permeability based on in silico models. The first prototypes of the Fraunhofer device will be available for study in vitro and in an animal model later this year. Potentially, this device will have broader applications, including use in diagnostics and therapeutics, as well as to guide drug formulation [164]. 

### 3.2. Formulation and Permeability

#### 3.2.1. Excipients and Permeability—Separating Effect from Artefact

The importance of excipient effects on drug permeability has been much discussed, and guidance on the subject was published in the International Council for Harmonisation of Technical Requirements for Pharmaceuticals for Human Use (ICH) “M9 guideline on biopharmaceutics classification system-based biowaivers (Step 5)” in 2020 [26,36]. M9 is a notable accomplishment and the first harmonized allowance of a Biopharmaceutics Classification System (BCS)-based regulatory relief, including in Japan.

M9 indicates that when there is a difference in excipient(s), BCS-based biowaiver applications should justify why the excipient differences will not impact the rate and extent of drug absorption and include mechanistic and risk considerations. M9 provides decision trees to facilitate such analysis and specifies that possible effects can occur via impacts on solubility, gastrointestinal motility, transit time and intestinal permeability, including transporter mechanisms. Sodium lauryl sulfate is exemplified as an excipient that may affect absorption.

M9 Annex II provides guidance on assessing possible excipient effects when excipients in the reference and test formulations differ. Excipient risk should consider, in a mechanistic fashion, excipient quantity, possible mechanisms via which the excipient may impact absorption and drug substance absorption properties (e.g., mechanism and extent of absorption). M9 states that Class III drugs have greater risk of excipient effects than Class I drugs. Hence, M9 requires more restrictive excipient considerations for Class III biowaivers than for Class I biowaivers.

##### Human Studies That Test for Excipient Effects on BCS Class 3 Drug Permeability

M9 indicates that drug permeability class (i.e., high or low) should be preferentially based on human pharmacokinetic studies. Presumably, human studies of excipient risk merit high reliability in assessing for possible excipient effects in a BCS-biowaiver application. M9 implies the need to consider excipient risk in a mechanistic fashion, since a BCS biowaiver application is very unlikely to contain a combined study of the excipient and drug substance in question. Class III biowaivers will almost certainly rely on external literature considerations of excipient risk [165]. Two such studies are discussed here.

Vaithianathan et al. conducted a series of bioequivalence studies in humans using two Class III drugs: cimetidine and acyclovir [166]. In studies with 14 common excipients, they concluded that 12 excipients need not be “qualitatively the same and quantitatively very similar” to the reference in a BCS-based biowaiver application. The 12 common excipients were sodium lauryl sulfate (SLS), sodium starch glycolate, corn starch, dibasic calcium phosphate, colloidal silicon dioxide, lactose, crospovidone, povidone, pregelatinized starch, steric acid, magnesium stearate and croscarmellose sodium. Large quantities of each excipient were tested. Meanwhile, the results of capsules containing both hypromellose (HPMC) and microcrystalline cellulose (MCC) failed the C_max_ criterion for bioequivalence (BE). It was speculated that MCC was an unlikely reason for high C_max_ and further studies were needed to investigate possible effects of these large amounts of MCC and HPMC. The limitations of C_max_ as a BE metric have been also discussed [165,167,168].

More recently, Metry et al. investigated potential effects of polysorbate 80 on active and passive intestinal drug absorption in humans [169]. Polysorbate 80 is a surfactant, an excipient class that has been mentioned in BCS guidance with regard to potential excipient concerns. A pharmacokinetic study in humans assessed valacyclovir, chenodeoxycholic acid (CDCA) and enalaprilat. Valacyclovir is a peptide transporter 1 (PepT1) intestinal substrate, CDCA is an apical sodium bile acid transporter (ASBT) intestinal substrate and enalaprilat exhibits very low passive permeability. The surfactant did not inhibit PepT1 or ASBT and did not increase enalaprilat absorption.

##### Rationale for Discordance between In Vitro and In Vivo Excipient Effects on BCS Class 3 Drug Permeability

M9 indicates that the drug permeability class can also be assessed using Caco-2 monolayers. An in vitro permeability method can be used when human studies (e.g., absolute availability, mass balance) do not exist or are conducted in a manner that does not allow class determination (e.g., insufficient collection of drug in mass balance study). Several in vitro studies have been performed to measure excipient effects on drug permeability.

M9 further indicates that Caco-2 permeability results should be considered in light of any human pharmacokinetic data. Presumably, given that Class III biowaivers will almost certainly rely on the external literature considerations of excipient risk, in vitro studies used to measure excipient effect on drug permeability should be considered when available and in light of any human pharmacokinetic data of a possible excipient effect on permeability. Hence, a combined examination of in vitro excipient effects and in vivo excipient effects will often be attempted for Class III drugs.

What should be the standard? Should in vitro always be assumed to be correct? Should in vivo always be assumed to be correct?

There are eight potential combinations of concordance and discordance between in vitro and in vivo results [167]. For example, if two products are truly bioequivalent, the four possible outcomes are that in vitro and in vivo are both correct, both are incorrect, only in vitro is correct and only in vivo is correct. Discordance between in vitro and in vivo results reflects the type I and type II errors of each approach. It merits recognition that both in vitro and in vivo methods suffer type I and type II error potential. For example, Rege et al. reported a false-positive outcome in 10% of all excipient effect studies [170], while C_max_ is a basis for type II error in bioequivalence studies [168].

It appears that Caco-2 monolayers can be expected to frequently over-predict in vivo effect of excipients, given differences between Caco-2 studies and human in vivo conditions. Caco-2 monolayers lack mucous and are a single monolayer, whereas the human intestine secretes mucus and, thus, presents a much greater physical barrier than the Caco-2 monolayer. Direct drug exposure effects (e.g., insult to cells) are more likely in Caco-2 monolayers than in in vivo tissue. Also, drug dilution and residence time effects in vivo results in a lower drug exposure of the absorbing surface to the drug than in Caco-2 monolayers. Overall, Caco-2 cells can be expected to be more susceptible to membrane disruption.

The literature supports this general trend of in vitro methods being overly sensitive. Parr et al. examined several excipients in studying Class III drugs [171]. Indeed, 0.1 mg/mL OF SLS increased Class III drug permeability across Caco-2 cells in spite of in vivo observations indicating no SLS effects [166]. Polysorbate 80 increases the cell membrane fluidity of Caco-2 cells, which presumably is a basis for the surfactant to inhibit PepT1 functioning in Caco-2 cells [170]. However, polysorbate 80 does not inhibit PepT1 in vivo or cause intestinal membrane disruption [169].

#### 3.2.2. Permeation Enhancers—Where Are We?

Orally delivered peptides must overcome several barriers, such as the acidic pH of the stomach, proteolytic enzymes, the mucus layer and the intestinal epithelium, in order to reach the systemic circulation. Strategies to overcome these hurdles include the use of enteric coatings, protease inhibitors, nanoparticles and medical devices [172,173]. An increasingly popular formulation approach is to include a permeation enhancer (PE) to aid the transport of the peptide across the intestinal epithelium paracellularly, transcellularly or a combination of both options [174]. PEs may interact with the lipid membrane of the cell, fluidizing it and allowing the peptide to enter the cell and cross transcellularly. Alternatively, PEs may work paracellularly by transiently opening the tight junctions between intestinal cells, allowing the peptide to move between the cells. Currently there are two formulations approved for the oral delivery of peptides that include a PE.

Rybelsus^®^ (Novo Nordisk, Bagsværd, Denmark) is an oral semaglutide tablet used to treat type-2 diabetes, which includes sodium salcaprozate (SNAC, 300 mg) as a PE [175,176]. Semaglutide is a glucagon-like peptide-1 receptor agonist (GLP-1 RA), modified to facilitate a longer half-life. SNAC enhances the transport of semaglutide transcellularly across the gastric epithelia and protects the semaglutide by creating a higher local pH [177]. In the presence of SNAC, semaglutide is in its monomer form, which can more easily cross the epithelia. It is important to note that even with all of these strategies, the formulation has a bioavailability of ~1%. 

Other pharmaceutical companies, such as AstraZeneca and Eli Lilly, are also interested in developing different GLP-1 analogues and delivering them orally. Astra Zeneca have specifically developed MEDI7219 for oral delivery [178]. The peptide was modified using amino acid substitution to make the peptide less vulnerable to proteases, and its potency was increased via the lipidation of the peptide backbone. An in vitro screen was carried out to select the best PE. PEs tested included sodium caprate (C10), different bile salts and SNAC. This screen identified a novel combination of the PEs, sodium chenodeoxycholate (NaCDC) and propyl gallate (PG). In a dog model, an enteric coated tablet containing 20 mg of MEDI7219 and 300 mg of PE (100 mg NaCDC and 200 mg PG) had a bioavailability of 6%. Eli Lilly formulated an acylated glucagon-like peptide-1/glucagon co-agonist peptide with a PE and a protease inhibitor for oral delivery [179]. The screen of PEs found sodium caprate, relative to other Pes, such as SNAC and lauroyl L-carnitine, to be the most effective PE when co-administered with the protease inhibitor soybean trypsin inhibitor (SBTI). In a mini pig model, when orally administered in enteric coated capsules, acylated peptide (25 mg) blended with C10 (500 mg), EDTA (150 mg) and SBTI (125 mg) achieved an oral bioavailability of 1%.

The second formulation on the market that contains a PE is Mycapssa^®^ (Amryt Pharma, Dublin, Ireland). Mycapssa^®^ consists of a capsule containing an oily suspension for the oral delivery of octreotide, which is a somatostatin analogue for the treatment of acromegaly [180,181]. The capsule contains sodium caprylate (C8) as the PE, along with other surfactants such as polysorbate 80 and glycerol mono/tri caprylate. The bioavailability achieved with this formulation is ~0.8%.

An orally delivered leuprolide to treat endometriosis, Ovarest^®^, is currently in clinical trials using Peptelligence^®^ technology (Enteris BioPharma, Boonton, NJ, USA). Previous versions of this technology comprised an enteric coating, citric acid as a pH modulator and acyl carnitines or bile salts as PEs [182]. A phase 2 clinical trial in healthy volunteers has been completed with promising results [183,184] and a trial is currently being carried out in women with endometriosis [185].

Labrasol^®^ ALF (caprylocaproyl polyoxyl-8 glycerides), a lipid-based non-ionic surfactant that contains C8 and C10, has also been investigated as a PE. In a rat intra-jejunal instillation model, Labrasol^®^ was shown to increase the relative bioavailability of insulin to 7% [186]. Merck screened tricyclic peptide PCSK9 inhibitors and found that when compound 44 was formulated with Labrasol^®^ (30%) and delivered orally to cynomolgus monkeys, a bioavailability of 3% could be reached [187]. While low, this bioavailability was sufficient to reach target levels in the blood. 

Realistically, the bioavailability achievable for a peptide is ~1–5% in the presence of a PE, and high concentrations of PE are needed to achieve this level. To improve on this goal, a number of strategies are being investigated, such the use of PEs in combination with nanoparticles [188]. It has been shown that some nanoparticles themselves can act as PEs [189]. Berg et al. designed an intestinal administration device that contained mini tablets made of sodium caprate and the peptide MEDI7219 [190]. The device was designed to release the tablets in contact with the wall of the intestine. While the device did not increase bioavailability more than enteric coated capsules, it did reduce peptide plasma concentration variability. It has been suggested that the release kinetics of formulations containing PEs could be controlled e.g., releasing the PE in two phases or the sustained released of the PE and drug over time, to improve the effects of the PE [191].

There is a need for more physiologically relevant in vitro models to screen intestinal PEs [192,193]. This development would improve the in vitro–in vivo correlation (IVIVC). A study carried out with sucrose laurate (C12) showed that the effective PE concentration in vitro was 1 mM, but in an intra-jejunal instillation model, it was 25–100 mM [194]. The GI Tract-Tissue Robotic Interface System (GI-TRIS), a high throughput screening tool using porcine intestine, has been developed, and it identified polyethyleneimine as a potentially suitable PE [195]. This PE was able to deliver a 11.3-fold increase in the oral bioavailability of oxytocin in vivo in a large animal model. Another model being investigated is the intestinal organoids generated from minipig tissue [196].

### 3.3. Colonic Absorption

Colonic absorption after oral administration of a drug/drug product is of interest in cases when a high dose–low solubility active pharmaceutical ingredient (API) is administered and absorption is incomplete in the small intestine, an extended (prolonged) release dosage form is administered or the product targets the colon for local action [197]. For a drug to be quantitively absorbed from the colon, three aspects need to be considered: (1) sufficient permeation across the colonic epithelium, (2) sufficient dissolution of the drug in the special environment of the colon (low volumes of free fluid, presence of microbiota, etc.) and (3) sufficient stability in colonic fluids.

The emphasis to date has been on the physicochemical characterization of the environment in the upper intestinal lumen of healthy adults, as the oral drug absorption is usually expected to be complete in the upper small intestine. However, differences in the luminal environment between the upper small intestine and the lower intestine may impact the performance of orally administered drug products, which deliver drugs during residence in the lower intestine. 

Modern imaging techniques (e.g., telemetric capsules, magnetic resonance imaging, etc.) offer some options to study the fate of orally administered drug products and have been successfully applied to investigate the pH and the fluid volume with little or even no bowel preparation [156,198]. In addition, a well-defined protocol for direct sampling from the lower intestine with minimal effects on its physiology under conditions to which drugs/drug products are exposed during BA/BE studies in healthy adults has been proposed [197] and applied in older adults [199].

Although roughly 1–2 L of intestinal contents are transferred into the large intestine every day, from which around 200 mL/day are excreted via the feces. Schiller et al. showed that the luminal fluid volume in the colon is relatively small, with measured values in the range of 1–44 mL in the fasted state and 2–97 mL in the fed state [198]. These volumes primarily exist in several fluid pockets located in the ascending colon and the descending colon. Due to the limited free water volume in the transverse colon [198], the regions of interest with regard to drug/dosage form performance in the lower intestine are the distal ileum, the cecum and the ascending colon. In the aspiration studies, contents were collected from the ascending colon over a period of approximately 10 min, and the volume of the contents in the ascending colon was approximately 23 ± 8 mL in the fasted state and 30 ± 11 mL in the fed state [200], with substantially lower volumes in the cecum (5 ± 2 mL in the fasted state and 8 ± 3 mL in the fed state) [197]. During colonic transit, the viscosity of the contents increases, while the volume of free fluid decreases. Consequently, the conditions for drug dissolution in the colon are relatively poor. This issue is of particular concern in the development of colon-targeted dosage forms, given the assumption that only dosage forms in contact with fluid can release the drug and noting that fluids are also needed to enable drug absorption by bringing the dissolved drug into contact with the absorptive surface of the intestinal tract [201]. 

**Figure 9:**
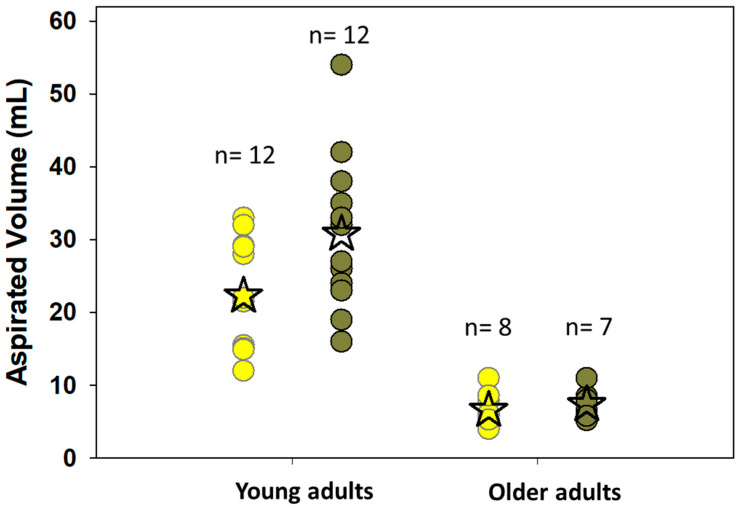
Individual values for the aspirated volumes of the contents of the proximal colon of young adults and older adults measured after overnight fasting (light yellow circles) and 5 h after a standard high-calorie, high-fat meal (dark green circles). *n* is the number of subjects, and ✩ denotes the mean value [199,200].

It is interesting to mention that in older adults (65–74 years of age), aspirated volumes from the distal ileum and the cecum were found to be even lower than those recovered from young adults (Figure 9) [199]. The macroscopic evaluation of the nature of contents in the proximal colon of older adults indicated that the fraction of contents adhering to the mucosa and, therefore, not amenable to aspiration during colonoscopy was substantial (65–90% of total contents), dissimilar to observations in young adults. The adhesion of water to the mucus may, thus, explain the difference in reported volumes of aspirated fluids in the two subject groups. The implications of these observations on drug release/dissolution from drug products in the lower intestine, as well as drug transport kinetics towards the surface of the mucosa, warrant further investigation [199]. 

Based on colonic volume data and the physicochemical characterization of colonic contents [197,200], a two-stage in vitro methodology for the evaluation of dissolution in the lower intestine using commercially available dissolution system and equipment has been proposed [202]. Based on this methodology, the dissolution can be studied via a single-compartment setup using a medium to simulate the environment in the distal ileum in Stage 1 and then, in Stage 2, a medium simulates the environment in the ascending colon in the fasted state or the fed state in all levels of simulation of biorelevant media [203]. In Level III simulating conditions, the viscosity effects on drug release were simulated using microcrystalline cellulose. The ratio of volumes in Stage 1 and Stage 2 was 1:5, which is similar to the average ratio of aqueous volumes in distal ileum and ascending colon of healthy adults observed in the clinical studies. Dissolution data were coupled with physiologically based oral absorption modeling to simulate the average plasma levels or the average absorption process, and the reliability of the modeling approach was evaluated based on previously collected data in adults. It was concluded that for immediate release products, pellets and products coated with pH-sensitive polymers (situations where stress effects are not expected to be of an issue), Level II or even Level I (if the API is not very lipophilic) biorelevant media in combination with the two-stage in vitro methodology seem to be adequate for the evaluation of dissolution in the lumen of the lower intestine of adults. For highly dosed low solubility APIs with long apparent terminal half-lives, the impact of absorption from the lower intestine on the plasma profile is very small. The simulation of actual drug particle dissolution in the lower intestine is not typically necessary for the adequate prediction of oral absorption from immediate release formulations containing discrete, dispersed particles of lipophilic drugs [202].

Aside from the volumes and viscosity of lower bowel contents, another factor that may limit colonic absorption is the bacterial degradation of APIs. In 2008, at least 30 drugs that have been commercially available were subsequently shown to be substrates of bacteria in the lower intestine [204]. Several in vitro systems have been developed with the aim of mimicking the periodic entry of fermentable substrates into the colon in order to study the bacterial degradation of APIs [204,205]. These in vitro setups do not always reflect the physiological bacteria density and the available volumes in the colon and their dynamics, and they need a long period of precultivation before building a stable culture. So, the biggest challenge in developing in vitro tools simulating the colonic bacterial environment seems to be creating a fermentation system that contains a physiologically relevant number and diversity of colonic bacteria. The simplest ex vivo techniques are the static batch cultures. These cultures can use human feces that are then placed into a suitable medium (saline or buffer solution). The drug is added in solution at time zero, and regular samples are withdrawn and quantified for the amount of drug and its metabolites. They are suitable for short incubation periods and are easy and flexible screening tools. It should be noted that a lack of justification of the level of dilution of stools and the questionable clinical relevance of the collected data may lead to the rejection of potentially useful therapeutic agents (false-negative decision) or the selection of problematic compounds for further development (false-positive decision) during the development phase [206]. 

Several years ago, the experimental conditions and the level of dilution of stools with normal saline were optimized based on clinically important degradation profiles of metronidazole and olsalazine in distal ileum and proximal colon of healthy adults [207,208]. Using optimized human fecal material to simulate bacterial degradation in the proximal colon [simulated colonic bacteria (SCoB)] consisting of 8.3% (*w*/*v*) human stools in normal saline has been proposed, and its usefulness in simulating nitro-, azo- and sulfo-reducase-related bacterial degradation activity in the lower intestine has been evaluated based on data from various model compounds [206,209]. Recently, it has been shown that the degradation half-lives generated ex vivo in SCoB could also be used in simulating the drugs’ performance and metabolism through in silico modeling [209]. The usefulness of this approach in the case of therapeutic agents, which are degraded by enzymes other than nitro-, azo-, or sulfo-reductases, and formulations targeting the lower intestine is worthy of further investigation.

Improving knowledge of the barriers to colonic absorption will enable better prediction of colonic absorption [210]. In vitro evaluation of permeability, together with colonic stability testing, is important to the early assessment of colonic absorption.

## 4. Conclusions and Perspectives

With the advent of ICH M9, methods of determining permeability have been harmonized across ICH and affiliated countries, making the regulatory landscape much easier for pharmaceutical companies to navigate in the area of permeability studies and their reporting requirements. However, it is generally recognized that Caco-2 monolayer systems may not adequately predict permeability in humans and tend to overpredict interactions with excipients. Thus, there is great interest in developing alternative approaches to predict human permeability, ranging from in silico approaches to 3D-human tissue scaffolds, and the field as a whole is rapidly evolving. 

On the clinical side, the limitations of absolute bioavailability studies and mass balance studies in terms of estimating permeability are widely recognized. Alternatives to jejunal perfusion studies are also sought, given that these methods are elaborate and difficult to extend to more distal regions of the human GI tract. In particular, for drugs that are administered in controlled release formulations and those that are targeted to the lower gut, methods to determine regio-specific permeability values are sorely needed. 

Recently, there has also been special interest in the ability of permeation enhancers to enable the oral administration of peptides, as this would circumvent the need for parenteral formulation of drugs in this increasingly important therapeutic category. Moreover, the advent of drugs that enable targeted protein degradation has led to growing interest in the application of permeation enhancers in the field of small molecules, as pharmaceutical scientists are looking for new ways to improve the oral absorption of these compounds because their chemical structure are often poorly permeable and poorly soluble.

## Figures and Tables

**Table 1:** Role of permeability in pharmaceutical development.

	Discovery (Pre-CN)	Development (Post-CN)		Clinical
	*Drug Substance*	*Drug Substance*	*Drug Product*	*Drug Substance/Drug Product*
*Physicochemical* *parameters*	Lipinski rule of 5 (MW, LogP/LogD, HBD, HBA), PSA aromatic rings, number of rotatable bonds, etc.; bRo5 requires additional property evaluations			
*In vitro/ex vivo tools (P_app_, P_eff_)*	PAMPA, cellular transport screening	Passive cellular permeability and active transport	Dissolution–permeation systems (e.g., biphasic dissolution, µFlux)	
*In vivo models* *(F, f_a_)*	Rodent PK	PK in higher preclinical species and food effect studies, extended release and regional absorption recorded in dogs		Human PK (F_abs_), bioequivalence, microdosing (F_abs_), perfusion studies
*Modeling* *(from P_app_, P_eff_,* *F to k_A_, f_A_, f_G_, f_H_,* *predicted dose,* *human PK)*	Static predictions: in silico tools, multiparametric scores (AB-), machine learning, artificial intelligence [34]	Dynamic models: PBPK modeling		

**Table 2:** Advantages, limitations and applications of two key in vivo permeability models used for drug permeability and absorption studies: single-pass intestinal perfusion (SPIP) and intraintestinal dosing/instillation.

Model	Advantages	Limitations	Applications
*SPIP*	Defined intestinal segment and surface area Allows direct drug permeability determination from luminal disappearance or indirect determination from plasma appearance Physiological regulation of gut functions is maintained Controlled luminal conditions Physiologically relevant permeability values	Slightly more labor intensive than the intestinal bolus model Tubing could cause non-specific binding of APIs	Determination of permeability independent of other absorption mechanisms Investigations of luminal, physiological and pharmaceutical effects on drug permeability Drug permeability can be studied at different physio-logical and pathophysiological conditions
*Intraintestinal dosing*	Very simple and efficient setup Minimal material use takes away risk of non-specific binding Can be used to investigate the entire absorption process, including dissolution, precipitation, gastric emptying kinetics, transit, etc. Useful for comparative formulation assessment	Permeability determination requires more assumptions than in the SPIP model Absolute *P_eff_* calculation less accurate due to lack of defined segment (relative assessment)	More generalized and formulation-related approach Investigation of absorption mechanisms as opposed to isolated permeability testing By comparing different formulation groups, different processes can be assessed more isolated (early preclinical formulation development)
